# Giant cabergoline-resistant prolactinoma in a man who presented with a psychotic episode during treatment: a case report

**DOI:** 10.1186/s13256-019-2071-2

**Published:** 2019-06-16

**Authors:** Luiz Augusto Casulari, Lucas Faria de Castro, Iruena Moraes Kessler, José Luiz Mendonça, Maria de Fátima Magalhães Gonzaga

**Affiliations:** 1 Endocrinology Service of the Brasilia University Hospital, Brasilia, DF Brazil; 2 Clinic of Neurology and Endocrinology, SCN quadra 1 bloco F Ed. America Office Tower, salas 1015 e 1016, Brasilia, DF 70711-905 Brazil; 3 Medical Clinic Service of the Brasilia University Hospital, Brasília, DF Brazil; 4 Institute of Cardiology of the Federal District, University Foundation of Cardiology, Brasília, DF Brazil; 5 Clinic of Radiology Vila Rica, Brasilia, DF Brazil

**Keywords:** Prolactinoma, Cabergoline, Resistance, Quetiapine, Mirtazapine

## Abstract

**Background:**

Prolactinomas are tumors of the pituitary gland that usually respond very well to treatment with cabergoline. Resistance to cabergoline is very rare, but when it occurs, it is a difficult problem to resolve if the tumor is inoperable.

**Case presentation:**

A 62-year-old white man was treated for a giant macroprolactinoma detected during investigation of a subacute subdural hematoma of the left frontal lobe. The patient was treated with cabergoline for 17 years with a dose ranging from 1.0 mg to 3.5 mg per week. We were not able to normalize his prolactin level, which initially was 14,992 ng/ml and ultimately 1754 ng/ml. The tumor significantly shrank during the follow-up period but persisted. The patient had cardiac valvulopathies that did not worsen. He had an ischemic stroke and developed a psychotic condition that was successfully treated by lowering the cabergoline and administering quetiapine and mirtazapine together. This regimen led to a small increase in the patient’s prolactin that returned to previous levels and remained as such until the last medical evaluation. The tumor continued to shrink and had a cystic degeneration in the last evaluation.

**Conclusions:**

Combined use of cabergoline with quetiapine and mirtazapine to treat a psychotic crisis may have contributed to shrinking the tumor in our patient because these antipsychotics have action mediated by growth factors that interfere with growth of pituitary tumors.

## Background

The dopamine agonists bromocriptine and cabergoline are the treatments of choice for individuals with prolactinoma. These options are based on expression of the dopamine receptor D2 found in pituitary tumor cells. When dopamine binds to these receptors, it lowers cell metabolism and prolactin transcription, leading to lower cell volume and cell death rates. The results obtained with treatments that use these agonists have been more beneficial than those obtained with surgery [1, 2].

However, some prolactinomas do not respond well to treatment with these dopamine agonists [3–5], and the mechanisms involved in prolactinoma resistance are not fully understood. Some studies have suggested the occurrence of changes in dopamine D2 receptors, such as lower counts of D2 receptors, lower expression of their coding genes, abnormal ratios of their short and long forms, and genetic polymorphism. Other factors that may play a role in the resistance of prolactinomas to dopamine agonists are abnormalities in growth factors, the extracellular matrix components, increased expression of genes involved in cell proliferation, and loss of suppressor genes at various loci [4, 5].

Increased cerebral dopaminergic tone due to the use of dopamine, bromocriptine, or cabergoline agonists may trigger psychiatric symptoms such as compulsive shopping behavior or sexual activity, gambling, and robbery; schizophrenia; psychoses; and intermittent explosive disorder [2]. The diagnosis of prolactinoma can be an incidental encounter when investigating some signs and symptoms related to other diseases [6–8].

We report a case of a patient whose macroprolactinoma diagnosis was established when he underwent magnetic resonance imaging (MRI) of the skull in a preoperative evaluation for drainage of a subacute subdural hematoma of the left frontal lobe. The patient developed psychotic symptoms at age 70, which was 7 years after initiating treatment with cabergoline and 1 year after having a stroke. The patient was followed from diagnosis until completing 17 years of treatment with cabergoline. Throughout treatment, the tumor was resistant to this dopamine agonist; the prolactin levels never normalized, and the tumor did show cystic degeneration but never disappeared.

## Case presentation

**Fig. 1:**
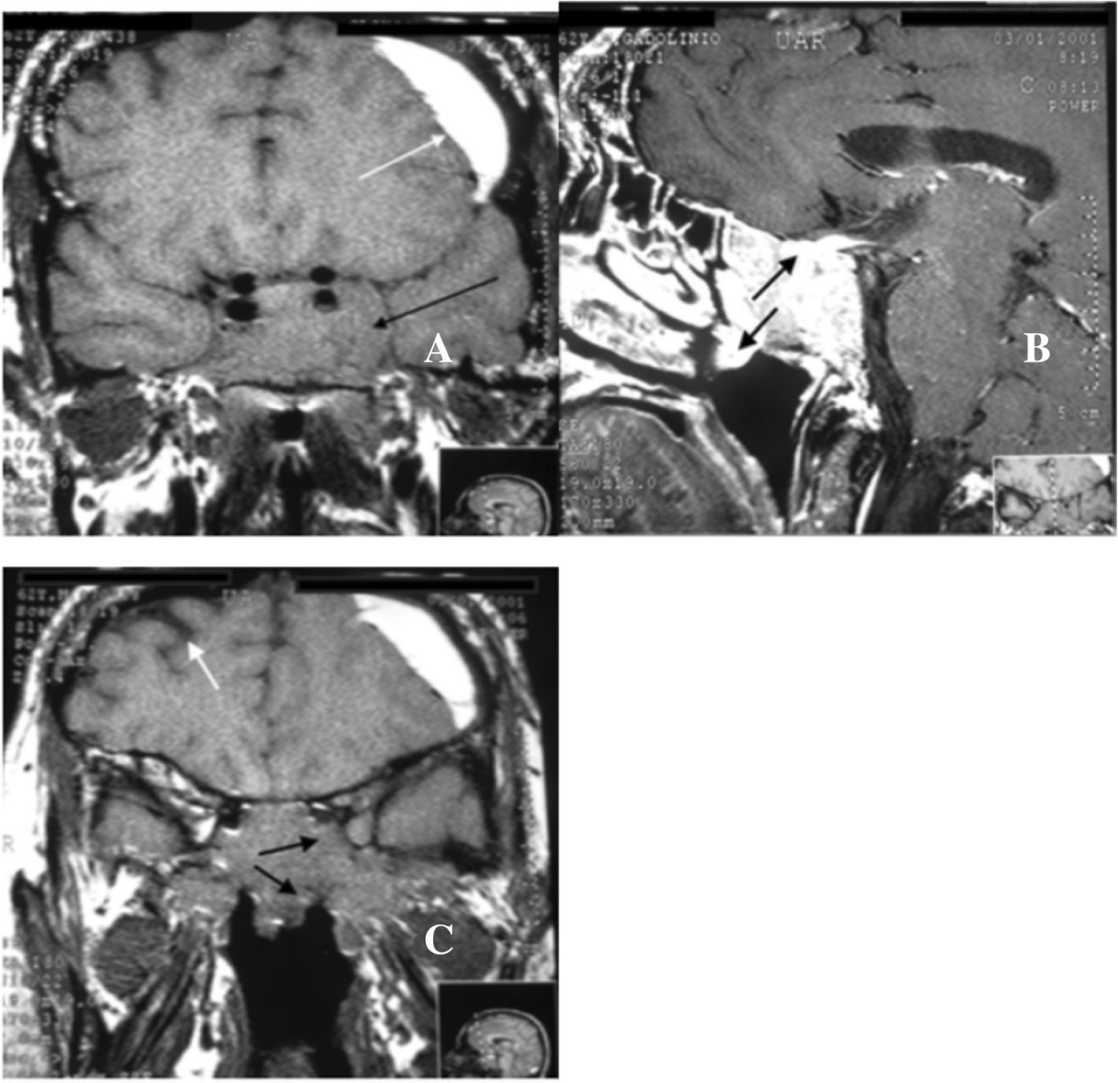
Magnetic resonance imaging of the sella turcica before starting treatment with cabergoline. **a** Coronal plane in T1-weighted image without contrast shows pituitary adenoma measuring 4.0 × 2.5 cm with total invasion of the sphenoid sinus and cavernous sinuses on both sides (*black arrow*). The left frontal subacute subdural hematoma is visible (*white arrow*). **b** Profile image in T1-weighted scan with heterogeneous contrast uptake showing total invasion of the sphenoid sinus with compression of the optic chiasm and invasion of the nasal area (*black arrows*). **c** Coronal plane image without contrast showing pituitary macroadenoma with invasion of the sphenoid and cavernous sinuses on the left (*black arrows*), in the right temporal lobe and senile atrophy (*white arrow*); discretely dilated ventricles and widening of the brain grooves can be seen

A 62-year-old white man first sought treatment for a subacute subdural hematoma of the left frontal lobe, for which he underwent trepanation. The neurological examinations done with contrast-enhanced MRI showed an elongated collection of hyperintense signals on T2, with peripheral enhancement, measuring 61 × 16 mm in the left frontal lobe, which characterized a subacute subdural hematoma. A solid tumor measuring 4.0 × 2.5 cm, occupying the base of the skull with total invasion of the sphenoid and the cavernous sinuses presenting suprasellar expansion was also observed. The tumor reached the optic chiasm and invaded the nasal area (Fig. 1a–c). A diagnosis of pituitary macroadenoma was made, but the occurrence of chordoma, metastasis, or cancer of the sphenoid sinus was also suspected.

The patient reported decreased libido and sexual impotence that had started 14 years earlier. He presented with no visual impairment and was a nonprofessional shooting competitor. He had astigmatism; his campimetry result was normal; and he did not complain of headaches.

Two-dimensional color flow Doppler echocardiography revealed a double aortic valve lesion with moderate stenosis, as well as concentric left ventricular hypertrophy with normal global and segmental systolic functions and left ventricular diastolic dysfunction. This pattern did not change throughout treatment.

At diagnosis, the patient had a prolactin level of 14,992 ng/ml (normal value < 17 ng/ml for males), follicle-stimulating hormone 0.5 IU/L (normal value up to 10 IU/L), luteinizing hormone 0.5 IU/L (normal value up to 9 IU/L), total testosterone 260 ng/dl (normal value 240 to 816 ng/ml), cortisol 25 μg/dl at 8 h (normal value 5.4 to 25 μg/dl), and 15 μg/dl at 16 h (normal value 2.4 to 13.6 μg/dl). The normal levels of cortisol secretion were preserved throughout treatment. During follow-up, the patient developed secondary hypothyroidism with thyroid-stimulating hormone (1.7 μIU/ml but free thyroxine 0.83 ng/dl, and replacement with 50 μg of levothyroxine was initiated. The patient’s complete blood count, electrolytes, urea, creatinine, glutamic pyruvic aminotransferase, glutamic oxaloacetic aminotransferase, γ-glutamyl transpeptidase, calcium, and phosphorus were normal during the 17 years of observation.

**Fig. 2:**
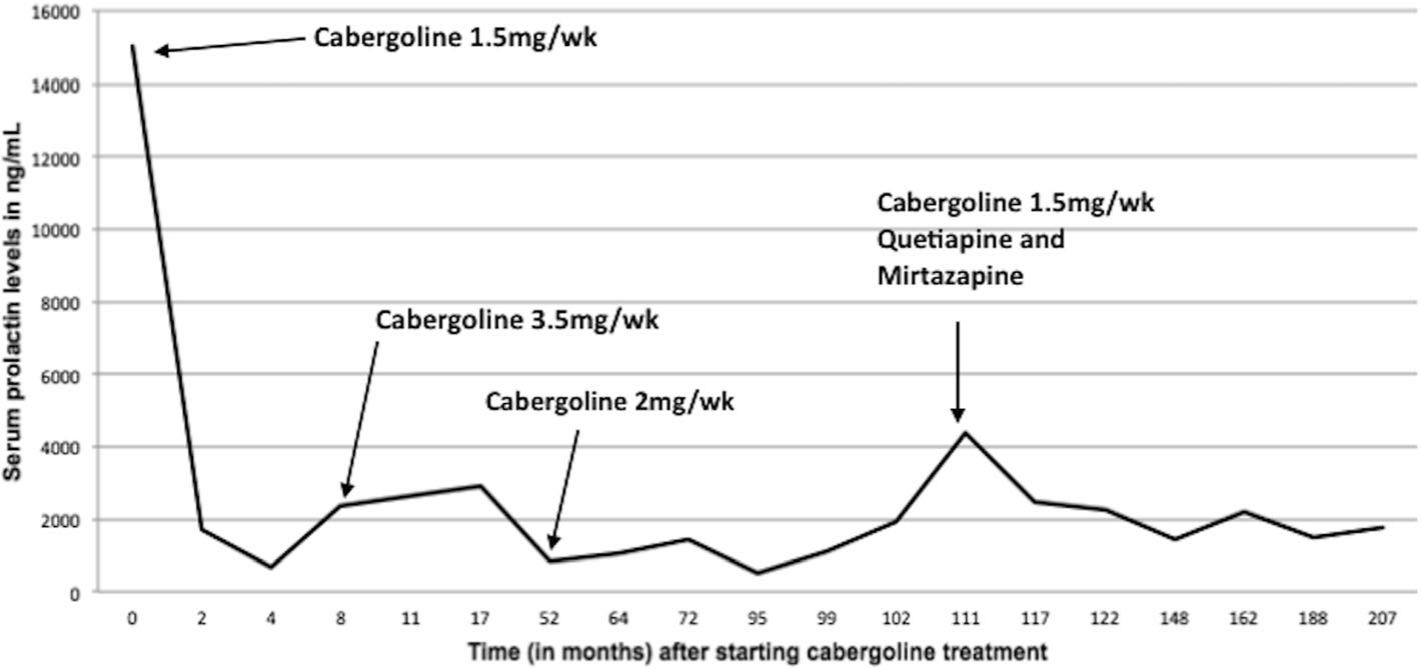
Prolactin levels in response to cabergoline use for 17 years. A drop in prolactin levels was observed with the cabergoline dose at 1.0 mg per week, but with no normalization of its levels. Levels were not normalized even at 3.5 mg per week, used for 44 weeks (from weeks 8 to 52). With the addition of quetiapine and mirtazapine to cabergoline, prolactin levels increased (111 weeks) and then decreased, without ever normalizing, until the last evaluation (1754 ng/ml), which was done 17 years after the start of treatment

Cabergoline treatment was initiated 2 months after diagnosis. The prolactin response is shown in Fig. 2. The prolactin level dropped from 14,922 ng/ml to 1717 ng/ml in the first 2 months of treatment with a 1.0-mg agonist. Nevertheless, the patient’s prolactin level remained high at 840 ng/ml even when taking a higher dose of cabergoline agonist of 3.5 mg per week for 48 months. At 111 months of treatment, quetiapine and mirtazapine were introduced, and the cabergoline was maintained, for treatment of psychotic conditions (see description below), causing a transient increase in prolactin that remained high until the last evaluation, 17 years after treatment was initiated.

After 4 months of cabergoline use, the prolactin level dropped to 646 ng/ml, but the testosterone level remained low (280 ng/dl), and sexual impotence persisted. Testosterone replacement was started, and the patient’s sexual activity normalized.

The patient presented with type 2 diabetes mellitus at first evaluation that was controlled with diet, metformin, and vildagliptin 50 mg. Glycated hemoglobin remained between 6.0% and 7.8% (normal value 4–6%). The patient also had high blood pressure. At the last evaluation, he was using indapamide 1.5 mg, bisoprolol hemifumarate 2.5 mg, amlodipine 5 mg, captopril 50 mg twice daily, and potassium chloride 600 mg twice daily. At age 67, and after 5 years of taking 2.0 mg of cabergoline weekly, the patient’s prolactin level was 1049 ng/ml. The patient underwent coronary angioplasty with stent placement due to unstable and progressive angina.

At age 69 and after 7 years of taking 2.0 mg of cabergoline weekly, the patient had a stroke. A computed tomographic scan showed right temporal intraparenchymal hemorrhage with ventricular flood. After neurosurgical interventions, the patient received phenytoin or carbamazepine for a few months.

One year after this event, he developed psychiatric alterations with persecutory delusion; he claimed that his neighbor was going to attack him. He also presented aggressive behavior with friends and family and demanded to have his firearm, which was used only for firing competitions, given back to him so he could defend himself, a behavior he had not presented previously. Initially, a 25-mg dose of quetiapine four times per day was used to control the psychiatric crisis. The dose was then changed to 100 mg once to three times daily as needed to control psychiatric symptoms; in the latter evaluation, he was taking 100 mg twice daily. Mirtazapine 30 mg every night was used throughout this period. Owing to the psychiatric outburst, the patient was maintained on a lower dose of cabergoline (1.5 mg per week) until the last evaluation.

**Fig. 3:**
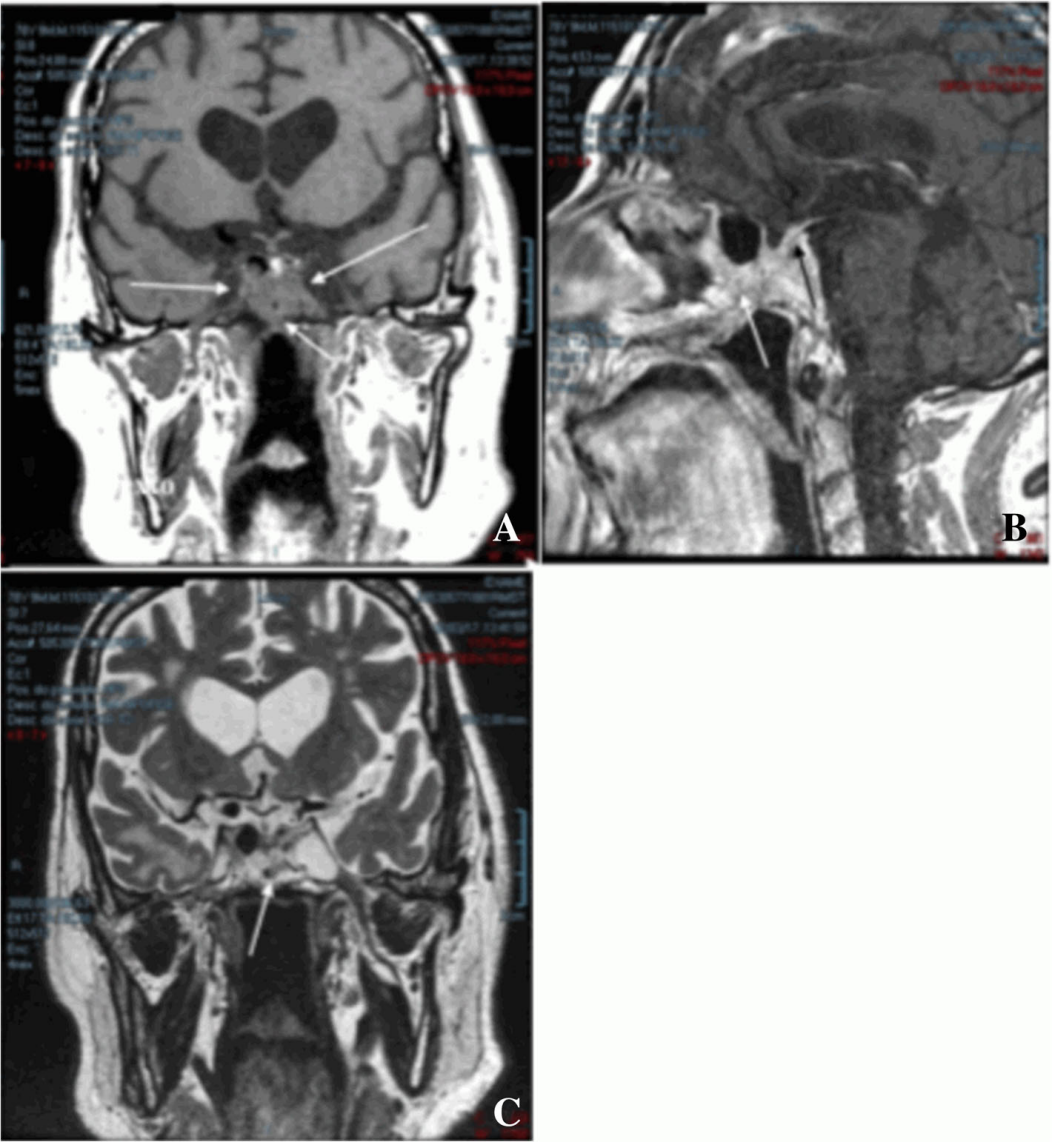
Magnetic resonance imaging of the sella turcica 17 years after starting treatment with cabergoline. **a** Coronal plane image with heterogeneous contrast uptake confirming the reductions of cavernous and sphenoidal sinus invasions (wh*ite arrows*). **b** Profile image with heterogeneous contrast uptake demonstrating invasion of the sphenoid sinus (*white arrow*) and with a pituitary stalk capturing contrast (*black arrow*). **c** Coronal plane image in T2 showing the cystic degeneration of the tumor (*white arrow*)

MRI evaluation of the tumor at 8 months and again at 17 months of taking 1.5 mg of cabergoline weekly showed no changes in tumor characteristics. The patient was lost to follow-up for 3 years; during this time, he used 3.5 mg of cabergoline weekly. MRI evaluation at 41 months of treatment showed partial regression of the adenoma, especially where it invaded the sphenoid sinus, but there was still invasion of the cavernous sinus on both sides but no suprasellar expansion. Figure 3 shows the images obtained in the last evaluations done at 17 years of treatment. Figure 3a shows a significant decrease in the pituitary adenoma with significant changes in the invasion pattern of the cavernous sinuses and the sphenoid sinus; the optical chiasm was downward. Figure 3b shows the tumor with heterogeneous contrast uptake and less invasion of the cavernous and sphenoid sinuses than when treatment began. Figure 3c shows heterogeneous uptake of contrast by the tumor with invasion of the sphenoid sinus and pituitary stalk. Figure 3d shows cystic degeneration of the tumor, hypersignal of the right temporal lobe, slightly dilated ventricles, and enlargement of the cerebral sinus.

## Discussion

The pituitary tumor of this patient was detected by MRI during investigation for signs and symptoms related to a subdural subacute hematoma. At the time, the patient stated that he had had sexual impotence for the last 14 years. His prolactin levels indicated that the tumor was a prolactinoma. The differential diagnosis of sellar masses includes adenohypophysis and neurohypophysis tumors, parasellar tumors, malignant tumors, malformation lesions, granulomatous and inflammatory lesions, and vascular lesions [6–8]. This patient had an enormous mass that invaded neighboring structures and the base of the skull with full invasion of the sphenoid and cavernous sinuses. For differential diagnosis, chordoma, metastatic cancer, and cancer of sphenoidal sinus were suggested. However, the patient’s prolactin level of 14,992 ng/ml confirmed the diagnosis of prolactinoma. The patient received a late diagnosis of prolactinoma, which reflects the delay among males in seeking medical attention when they present with complaints of sexual dysfunction.

The tumor presented by the patient at the time of diagnosis is considered giant because it had diameters equal to or greater than 4 cm. These tumors, which represent less than 4% of prolactinomas, are found more frequently in men in their 50s and are more aggressive in men than in women [2]. In a study of cabergoline-resistant prolactinomas in which 16.3% of giant tumors were found, 80% of them were in men [5].

Frequently, giant prolactinomas are associated with compressive symptoms in surrounding areas, such as the optic chiasm [2]. However, our patient, despite having this problem (Fig. 1), never had his visual acuity altered and could maintain his regular sporting activity of target shooting. It is very likely that the slow growth rate of the tumor allowed the optic chiasm and optic nerve to adapt to the compression caused by the tumor mass.

Treatment of giant prolactinoma is done with dopamine receptor agonists alone or in combination with surgery to reduce tumor volume, or with radiotherapy to prevent tumor growth [2]. The option of not subjecting the patient to surgery was made for several reasons: The tumor was giant, with invasion of neighboring structures, and it would not be possible to remove it completely; the patient was elderly, with associated diseases, mainly cardiac valvulopathies, which would make it dangerous for him to undergo tumor surgery. In addition, with monitoring with use of cabergoline, the tumor did not show that it was growing.

The goal of treatment of giant prolactinoma is to normalize prolactin levels and restore gonadal function [2]. However, intake of cabergoline for 17 years failed to control this patient’s prolactin levels, and testosterone replacement therapy was adopted throughout treatment. Tumor volume decreased only after many years of taking cabergoline, but the tumor did not fully disappear (Fig. 3). This behavior in relation to the use of high doses of cabergoline characterized the resistance to this agonist [3–5, 9–12]; it is more common in males and is associated with cavernous sinus invasion [5, 9]. In a multicenter study of 92 patients with cabergoline-resistant prolactinoma, 82.6% had macroadenomas, and the most striking features were that affected men were older than women and that males had more aggressive disease with invasion of the cavernous sinuses [5]. These characteristics in men were present in our patient.

Our patient developed psychotic symptoms at age 70, which was 7 years after initiating treatment with cabergoline and 1 year after a stroke. The use of cabergoline or bromocriptine is associated with the development of psychiatric symptoms [2, 13]. However, these side effects are usually triggered early in the first year of treatment with cabergoline [2]. Thus, it is not possible to establish that the psychotic condition the patient developed was triggered by the prolonged use of cabergoline, just as it is not possible to rule out that the dopaminergic agonist may have contributed to the onset of his psychiatric symptoms. On the other hand, it should be mentioned that delirium is a common complication after stroke [14, 15].

Because the onset of psychiatric symptoms may be related to higher doses of cabergoline [13], the dose of cabergoline was maintained at 1.5 mg per week until the last evaluation, which was 9 years after the onset of psychiatric symptoms.

The best treatment for psychiatric conditions is dopamine antagonists to treat the cerebral dopaminergic hyperactivity. The adverse effects of dopamine antagonists such as risperidone, ziprasidone, clozapine, olanzapine, paliperidone, haloperidol, and sulpiride are galactorrhea, menstrual alteration, reduced libido, osteoporosis, and metabolic syndrome [16]. It is possible that high levels of dopamine antagonists compromise the action of cabergoline to reduce prolactin levels. Therefore, it is worrisome when prolactin levels are high in psychiatric patients taking dopamine agonists [16].

The use of antidepressant medications that do not significantly interfere with prolactin secretion, such as olanzapine, quetiapine, and mirtazapine, is a good alternative in these cases. Quetiapine and mirtazapine were successfully taken by this patient until the last evaluation, 9 years after the onset of the psychiatric condition. These drugs are very effective in the treatment of psychiatric diseases and do not significantly interfere with prolactin secretion [16–18]. However, as shown in Fig. 3, a small increase in prolactin levels occurred shortly after starting treatment with either drug and while cabergoline was reduced to 1.5 mg per week to minimize its potential of triggering psychotic outbursts [2]. This strategy was effective, and the patient had no further psychotic crisis until the last evaluation.

Quetiapine has lower affinity for the dopamine D2 receptor. The drug has been successfully used to treat a woman with schizophrenia, who was a user of cocaine and alcohol and who had previously stopped treatment with risperidone because of the hyperprolactinemia triggered by this drug [19]. Mirtazapine differs from other antidepressants because it does not inhibit the reuptake of noradrenaline and serotonin but acts as an antagonist of the presynaptic α2 at postsynaptic 5-hydroxytryptamine 5-HT2 and 5-HT3 receptors and at histaminergic H1 receptors [20]. Thus, this special mechanism of action is associated with a hormone profile of decreasing cortisol and corticotropin (adrenocorticotropic hormone) secretion, but it has no action on prolactin and growth hormones [20, 21].

In a review, Chouinard *et al.* [22] showed that the use of first- or second-generation antipsychotics for more than 3 months may increase the density and function of dopamine D2 receptors. This increase may contribute to the occurrence of psychosis syndrome by hypersensitivity, which is characterized by rebound reactions of psychosis, tolerance to antipsychotic therapeutic effects, and tardive dyskinesia. Quetiapine, used in our patient, is one of the second-generation antipsychotics that most causes this syndrome. However, this syndrome was not observed in our patient, despite his using the drug for 9 years. It is possible that cabergoline may have played a role in preventing the development of the syndrome. It is also possible that the antipsychotic drugs and their action on D2 receptors may have contributed to the action of cabergoline in shrinking the tumor. These hypotheses require further investigation.

Interestingly, the effects of antidepressants are associated with quantitative changes in growth factors. Our patient’s use of mirtazapine and quetiapine for 9 years may have contributed to shrinking the tumor, even though the cabergoline dose was low. This is so because mirtazapine [23] and quetiapine [24] decrease the antidepressant actions associated with tumor necrosis factor-α (TNF-α). TNF-α level is increased in invasive pituitary adenomas [25] such as the one diagnosed in our patient. However, increased TNF-α associated with the metabolic syndrome and use of quetiapine has been reported [26].

On the other hand, as reviewed by Recouvreux *et al.* [27], transforming growth factor-β1 (TGF-β1) may be involved in resistance to dopamine agonists in patients with prolactinoma, with reestablishing TGF-β1 activities being a good strategy to treat these tumors. TGF-β1 decreases lactotrophic cell proliferation and prolactin production while also being involved with the role of dopamine-mediated inhibition on lactotrophic cells. It is expected for prolactinomas to have reduced TGF-β1 inhibitory activity. The lithium in the quetiapine lowers TGF-β1 levels [24], which may have contributed, at least in part, to the small increase in prolactin levels observed with the use of quetiapine.

It is possible that antidepressants could act differently on reducing the prolactinoma, with worsening prolactin production and improving tumor reduction. Discrepancies between the response to the dopamine agonist in relation to normalization of prolactin levels and reduction of tumor mass have been described [3].

Another aspect to consider is the possibility of heart valve damage due to prolonged use of cabergoline [2], as occurred with our patient. The patient already had double aortic injury at the start of treatment, but the tricuspid and mitral valves were preserved. In a long-term study of up to 5 years with cabergoline dose ranging from 0.25 mg to 5 mg per week and a cumulative maximum dose of 1260 mg, the prevalence of tricuspid and mitral regurgitation was not significantly elevated [28]. It has been suggested that echocardiography be performed in patients who have had audible cardiac murmur and have been treated for more than 5 years with doses of more than 3 mg per week of cabergoline or in those older than 50 who continue treatment [29]. Our patient had all of these conditions when treatment started. However, follow-up with echocardiography was done periodically. In any case, worsening of aortic valve lesions or the occurrence of a tricuspid or mitral valve injury was not observed, even when the patient was taking 3.5 mg of cabergoline per week.

## Conclusions

The treatment of our patient with prolactinoma, who had resistance to cabergoline, was difficult mainly because the patient was elderly and presented with important cardiomyopathy and because the tumor was invading the cavernous sinus. Surgery was not indicated. However, conservative treatment with prolonged use of cabergoline, combined with mirtazapine and quetiapine, may have contributed to cystic degeneration of the tumor, despite the fact that our patient’s prolactin levels never normalized.

## Abbreviations

5-HT: 5-Hydroxytryptamine
MRI: Magnetic resonance imaging
TGF-β1: Transforming growth factor-β1
TNF-α: Tumor necrosis factor-α
